# Supplementary material for: Mechanisms governing the pioneering and redistribution capabilities of the non-classical pioneer PU.1
Source: Nat Commun. 2020 Jan 21;11:402. doi: 10.1038/s41467-019-13960-2 (PMC6972792; doi:10.1038/s41467-019-13960-2)
Supplement: Supplementary file 7 — Source data [file 41467_2019_13960_MOESM7_ESM.zip › Source_Data/FigureS2/FigureS2B_MotifScanOutput/KmeansCluster11/homerResults/motif32.similar.html]

motif32

## Information for motif32

A
C
G
T
A
C
G
T
C
G
T
A
A
C
G
T
A
G
T
C
A
C
G
T
A
T
G
C
  
Reverse Opposite:  

T
A
C
G
C
G
T
A
A
C
T
G
C
G
T
A
A
C
G
T
C
G
T
A
C
G
T
A
  

|  |  |
| --- | --- |
| p-value: | 1e-18 |
| log p-value: | -4.152e+01 |
| Information Content per bp: | 1.926 |
| Number of Target Sequences with motif | 381.0 |
| Percentage of Target Sequences with motif | 11.73% |
| Number of Background Sequences with motif | 3267.8 |
| Percentage of Background Sequences with motif | 7.23% |
| Average Position of motif in Targets | 97.4 +/- 55.9bp |
| Average Position of motif in Background | 96.9 +/- 63.0bp |
| Strand Bias (log2 ratio + to - strand density) | -0.2 |
| Multiplicity (# of sites on avg that occur together) | 1.08 |
| Motif File: | file (matrix) reverse opposite |

### Similar de novo motifs found

|  |  |  |  |  |  |  |  |
| --- | --- | --- | --- | --- | --- | --- | --- |
| Rank | Match Score | Redundant Motif | P-value | log P-value | % of Targets | % of Background | Motif file |
| 1 | 0.954 | A T C G A C G T A C G T C G T A A C G T A G T C A C G T A G T C | 1e-16 | -38.891084 | 5.32% | 2.52% | motif file (matrix) |
| 2 | 0.918 | T C A G A G T C A C G T C G A T G T C A A G C T G T A C A C G T A G T C | 1e-16 | -36.949163 | 5.29% | 2.56% | motif file (matrix) |
| 3 | 0.875 | C G A T A C T G C G T A A T C G T C G A A G C T T C G A G T C A T C A G A G C T | 1e-14 | -33.251084 | 2.77% | 1.02% | motif file (matrix) |
